# Supplementary material for: Global Evaluation of Congenital Heart Disease-Associated Non-Coding Variants
Source: Res Sq. 2026 Jan 7:rs.3.rs-8429365. Preprint. [Version 1] doi: 10.21203/rs.3.rs-8429365/v1 (PMC12803342; doi:10.21203/rs.3.rs-8429365/v1)
Supplement: 1 [file NIHPPRS8429365V1-supplement-1.pdf]

Supplementary Figures

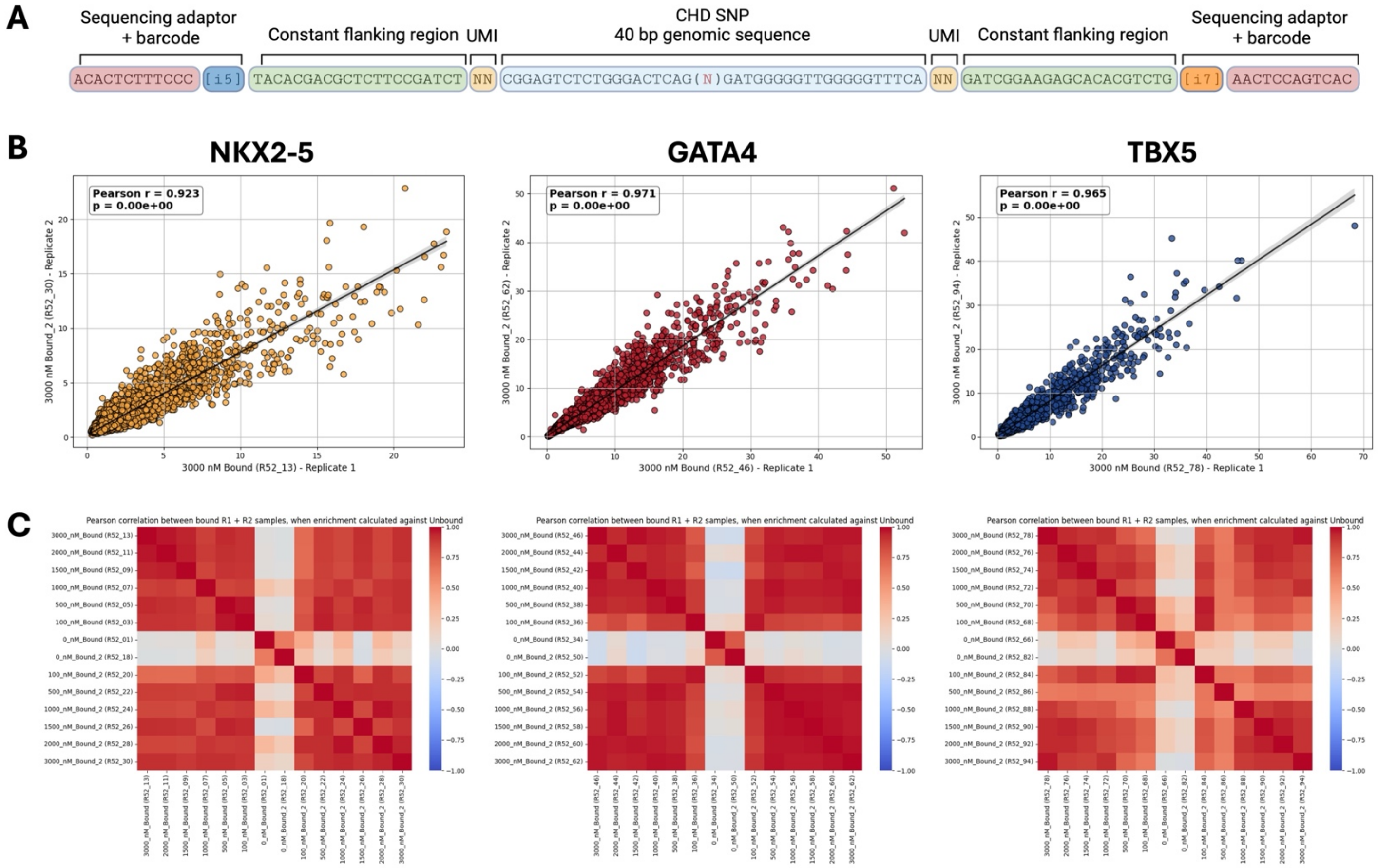

Supplementary Figure 1: SNP Bind-n-Seq library anatomy and replicate correlation. **A)** SNP Bind-n-Seq library sequence features, structures, and constant regions. **B)** Experimental correlation between replicates of NKX2-5 (left), GATA4 (middle), and TBX5 (right) at 3,000 nM. **C)** Correlation Matrix of Replicate Enrichment Values Relative to Unbound Conditions. Heatmap of the correlation matrix of NKX2-5 (left), GATA4 (middle), and TBX5 (right) enrichment across concentrations, relative to the corresponding Unbound condition in both replicates. Each cell represents the Pearson correlation coefficient between two concentrations, indicating the similarity in enrichment patterns.

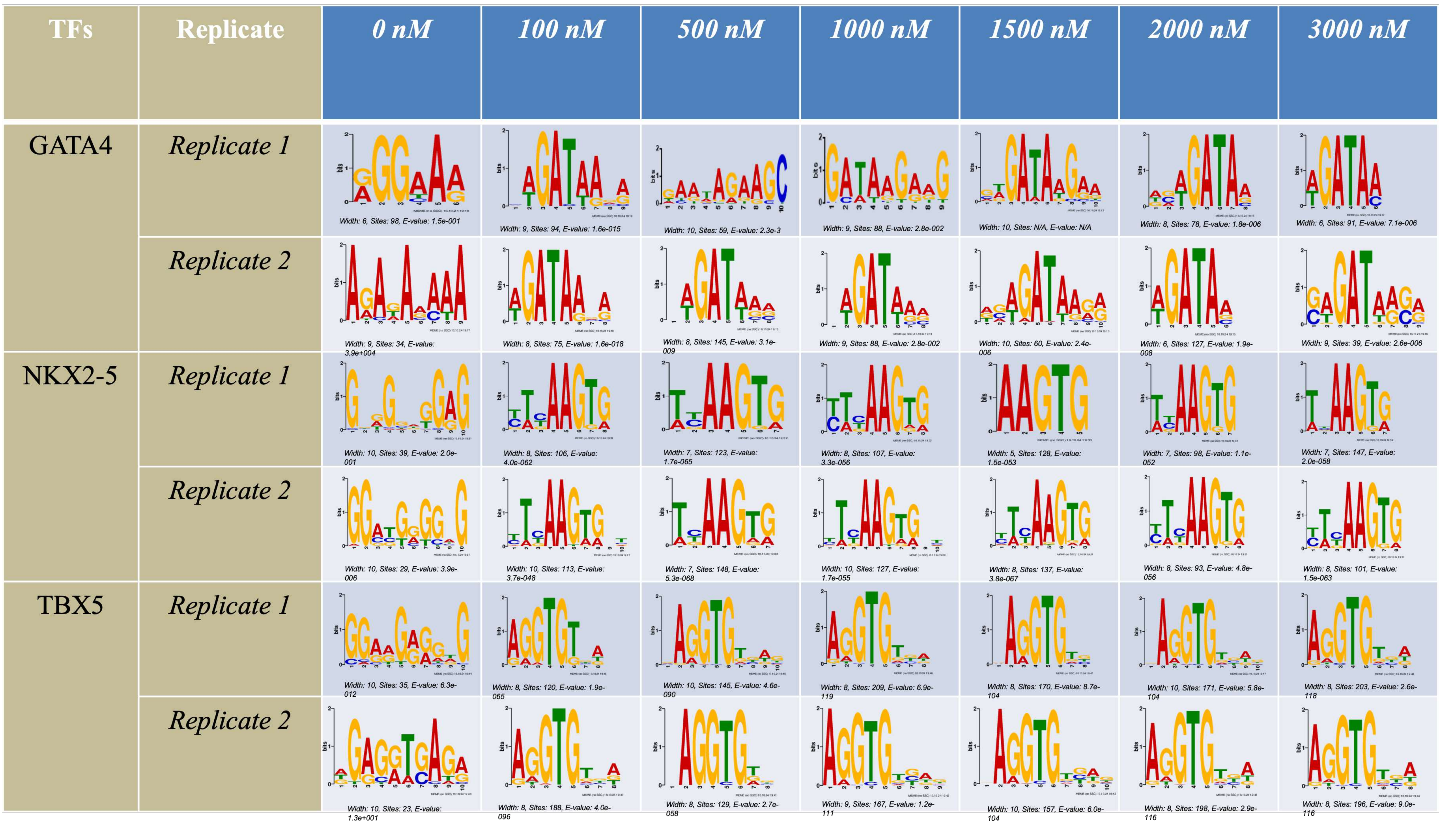

Supplementary Figure 2: TF motif enrichment across SNP Bind-n-Seq replicates and concentration points. Motifs were generated using MEME with the top 500 sequences with the highest K_A values.

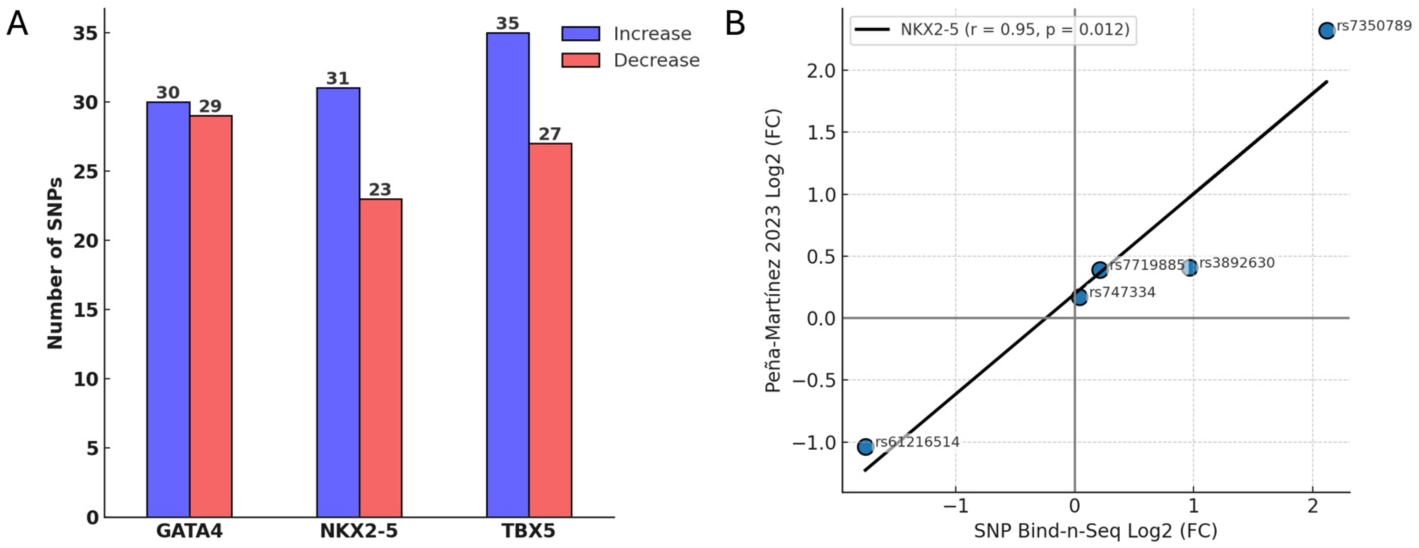

Supplementary Figure 3: Variants with differential allelic binding identified through SNP Bind-n-Seq. **A)** Number of variants with allelic binding for NKX2-5, GATA4, and TBX5. **B)** Fold change correlations of previously described variants for NKX2-5 binding affinity.

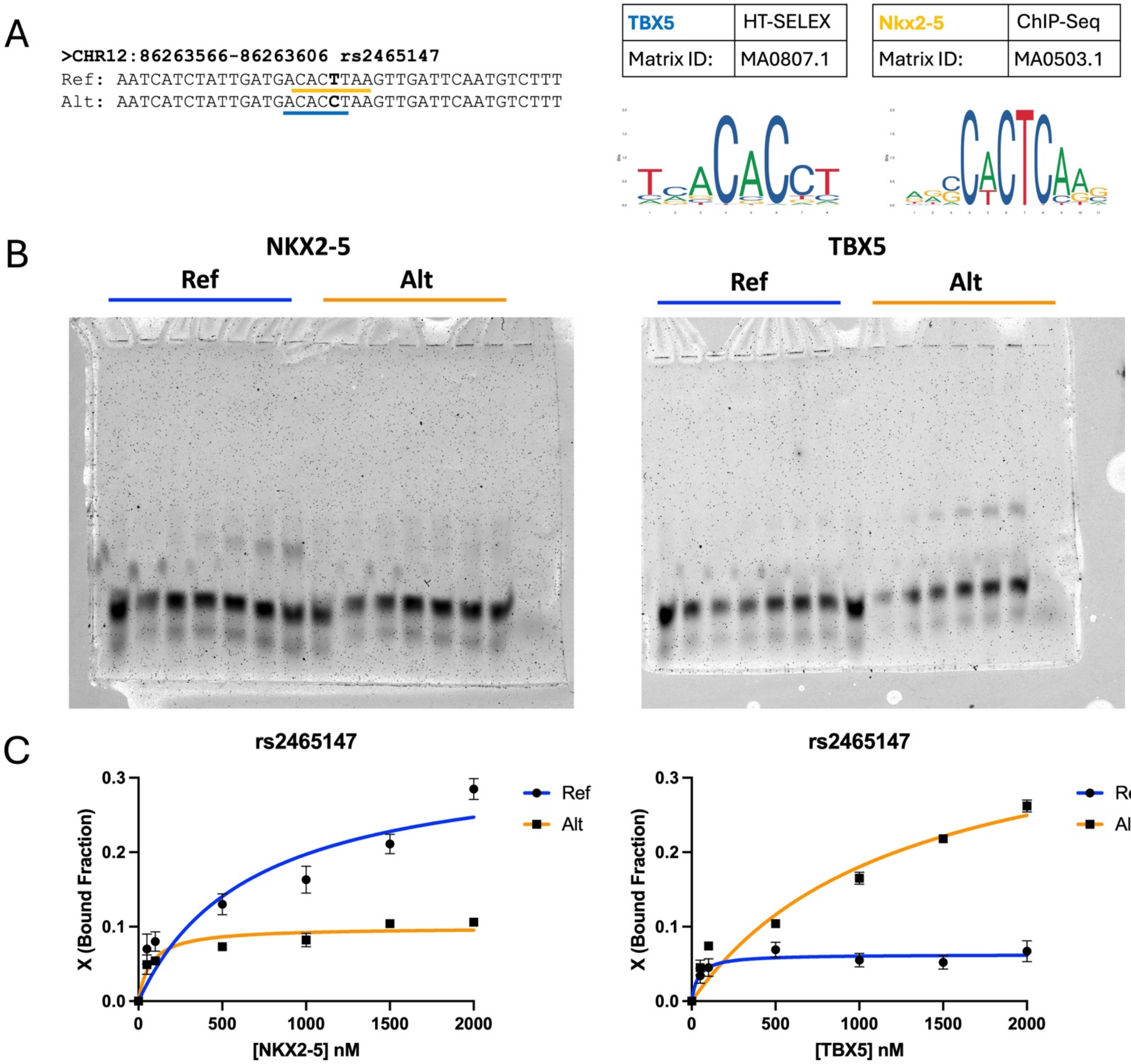

Supplementary Figure 4: In vitro validation of SNP Bin-n-Seq variant rs2465147. **A)** Reference and alternate sequences. TF binding motifs are highlighted in yellow for NKX2-5 and blue for TBX5. JASPAR motifs from NKX2-5 and TBX5 are displayed on the right. **B)** Electrophoretic mobility shift assay (EMSA) of rs2465147 for NXK2-5 (left) and TBX5 (right). **C)** Binding curves generated from EMSA of rs2465147 in triplicate.

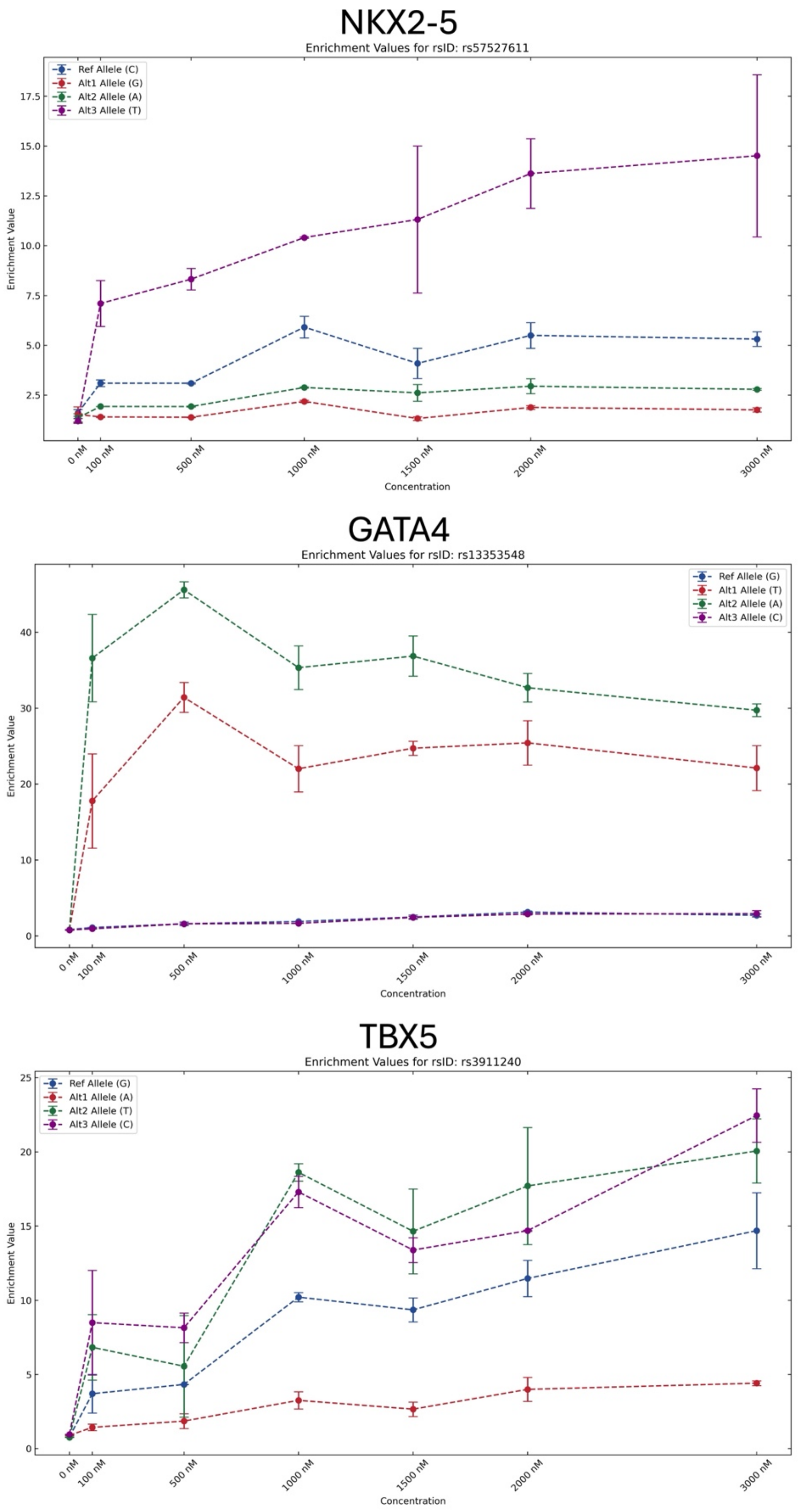

Supplementary Figure 5: Allelic enrichment curve of non-CHD-risk alternate alleles. Variants rs57527611, rs13353548, and rs3911240 are plotted for NKX2-5 (top), GATA4 (middle), and TBX5 (bottom) binding, respectively. Reference alleles (Ref) are represented in blue, and tag-SNP alleles from the GWAS catalog (Alt 2) are represented in red. Permutated alleles (alternate non-risk; Alt 2 and Alt 3) are represented in green and purple, respectively.

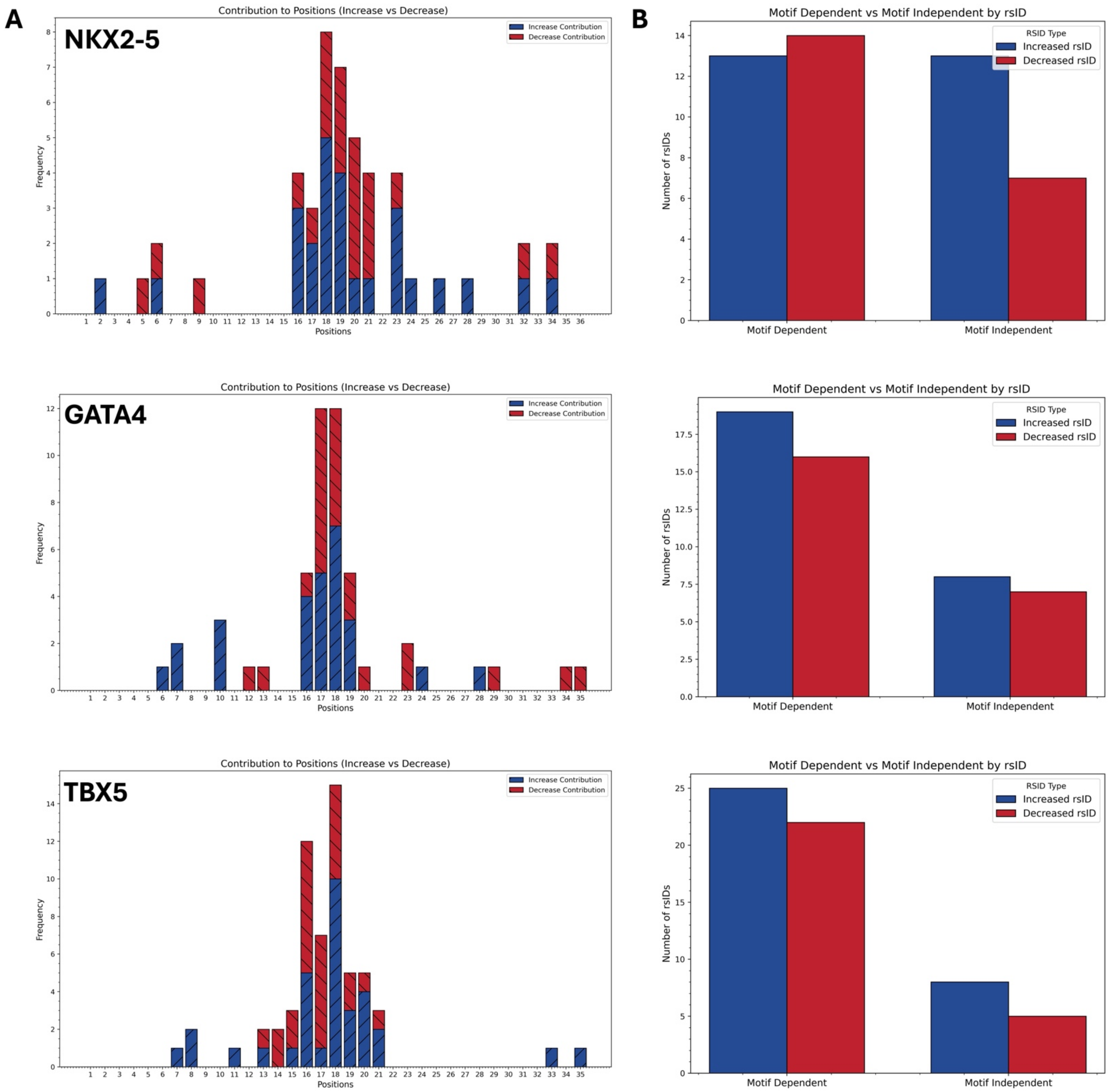

Supplementary Figure 6: Motif disruption analysis of variants with allele-specific binding. **A)** Binding motifs distribution of variants with allele-specific binding. X-axis represents the 40 bp window centered on the variant. **B)** Count of variants that directly create or disrupt TF binding motifs (motif dependent), adjacent to TF motif (motif independent). Variants creating binding motifs are represented in blue, whereas disrupting variants are represented in red.

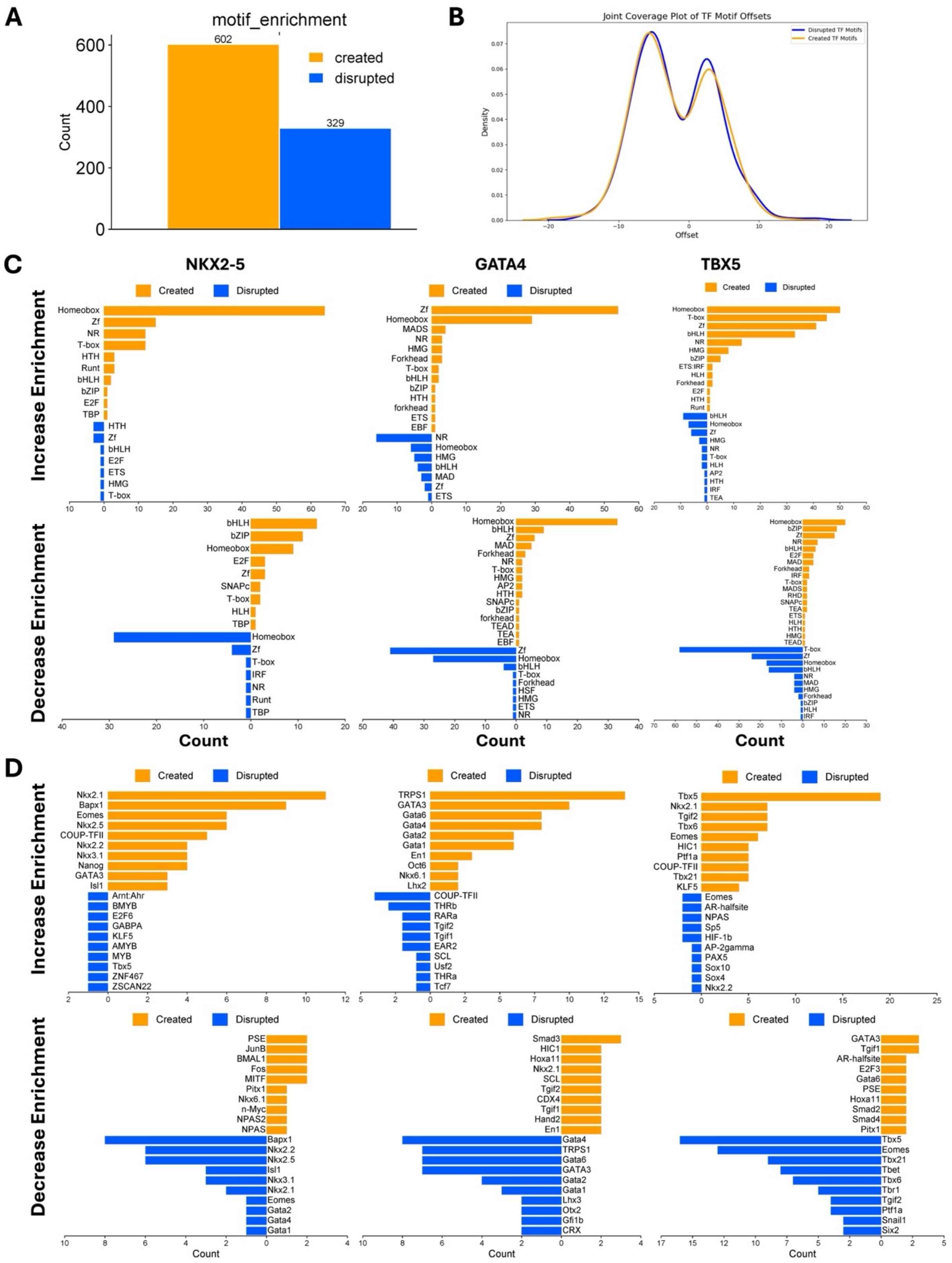

Supplementary Figure 7: Homer motif enrichment analysis of variants with allele-specific binding. **A)** Number of variants that created (orange) or disrupted (blue) TF binding motifs. **B)** Frequency of created and disrupted TF binding motifs relative to variant position (X = 0). **C-D**) Number of motif created or disrupted for **C)** TF families and **D)** specific TFs. Motif enrichment analysis are displayed separately for variants that increased or decreased binding affinity for NXK2-5, GATA4, and TBX5.

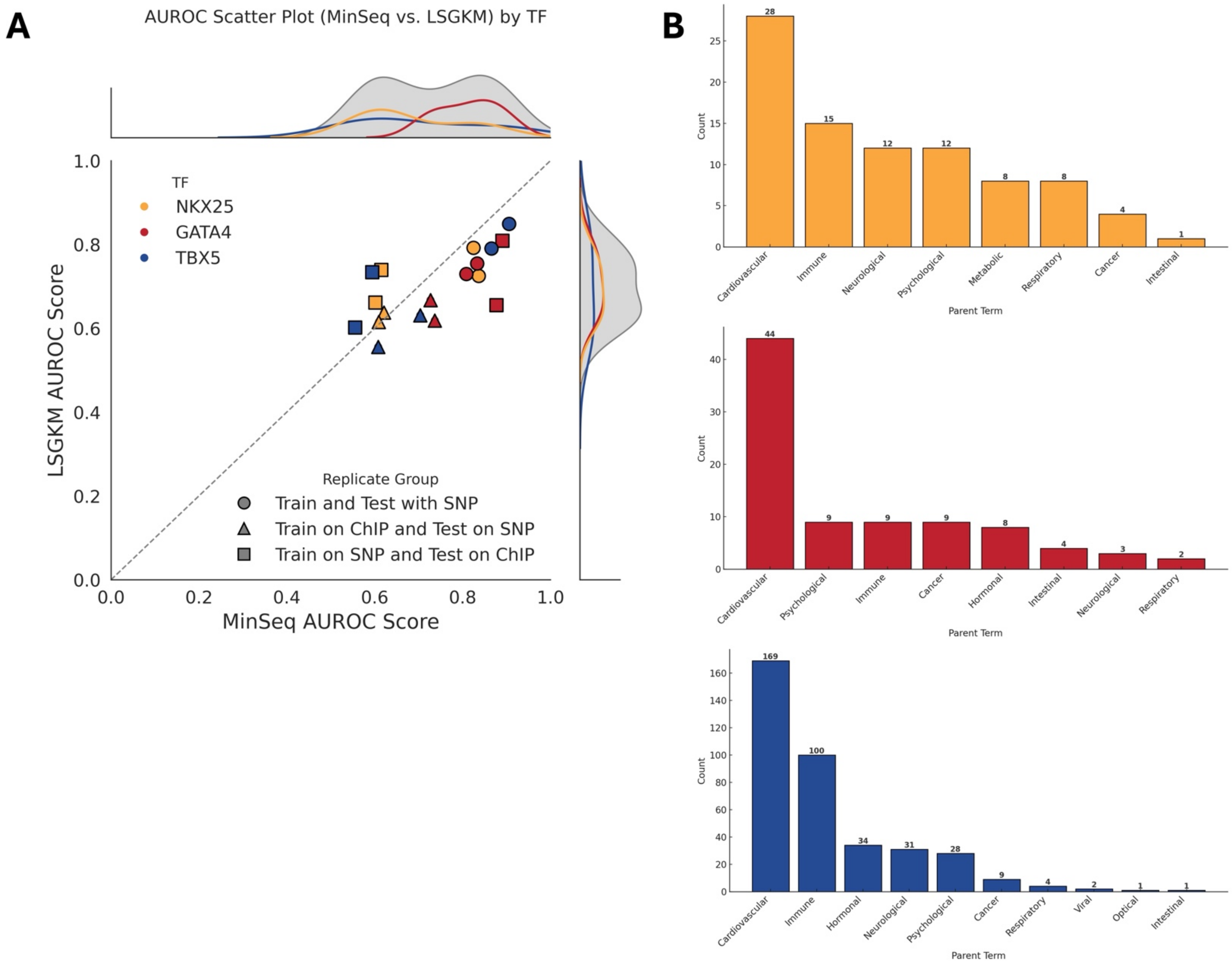

Supplementary Figure 8: Training predictive models with SNP Bind-n-Seq experimental data. **A)** Scatter plot comparing MinSeqChIP and LSGKM classifier performance for three transcription factors (NKX2-5, GATA4, TBX5), with AUROC scores shown on the x-axis (MinSeqChIP) and y-axis (LSGKM). Marker shapes indicate evaluation strategies: circles represent training and testing on SNPs, triangles indicate training on ChIP-seq and testing on SNPs, and squares indicate training on SNPs and testing on ChIP-seq. For each condition, two points are plotted because the same positive set was evaluated against two different negative sets: LSGKM-generated negatives and genomic negatives located 6,000 bp away from positives. In SNP→SNP evaluation, a 60–40 split is used for both classifiers, with 60% of the data (300 positives and 300 negatives) used for training and 40% (200 positives and 200 negatives) used for testing. In ChIP→SNP evaluation, training is performed on the top 1,000 ChIP-seq peaks and testing on 500 SNP positives (controls excluded). In SNP→ChIP evaluation, training is on 500 SNP positives (controls excluded) and testing on 1,000 ChIP-seq peaks. The dashed diagonal line indicates equal classifier performance, while marginal kernel density estimates summarize the overall distribution of AUROC scores. Points above the diagonal indicate superior LSGKM performance, whereas points below indicate better MinSeqChIP performance. **B)** Number of variants predicted to alter TF binding per disease parent term from the GWAS catalog. NKX2-5 is represented in yellow, GATA4 in red, and TBX5 in blue.

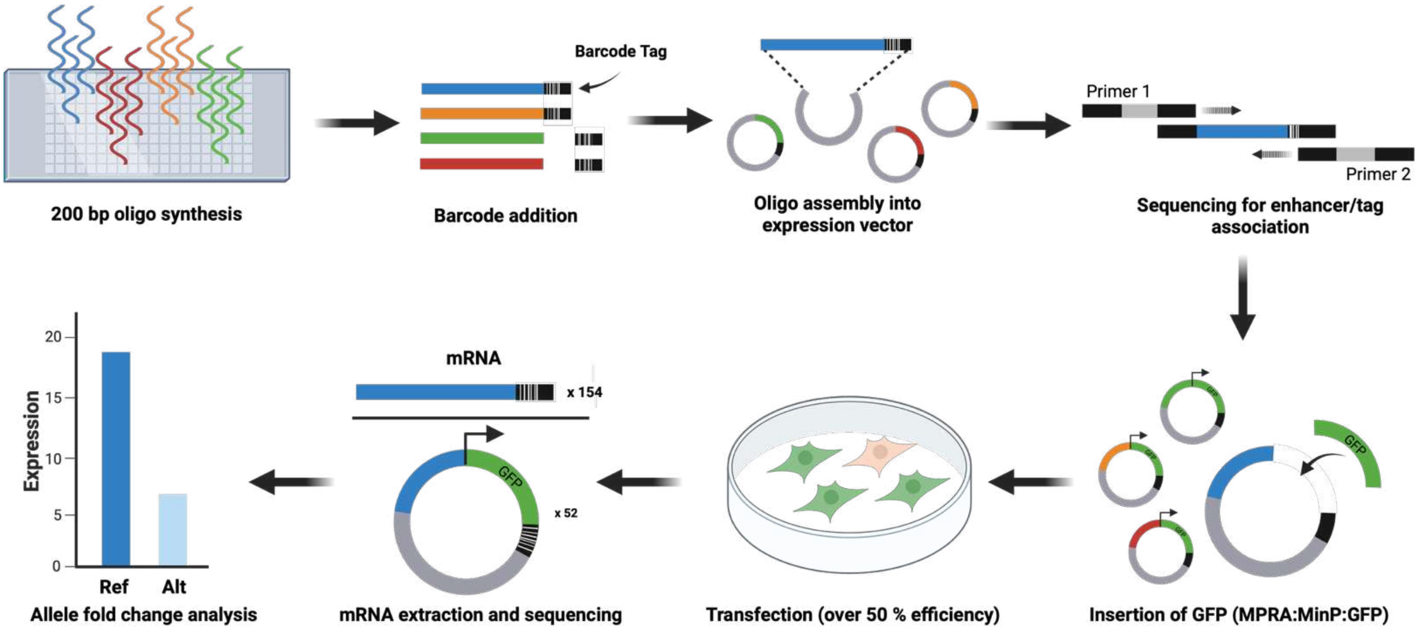

Supplementary Figure 9: MPRA workflow.

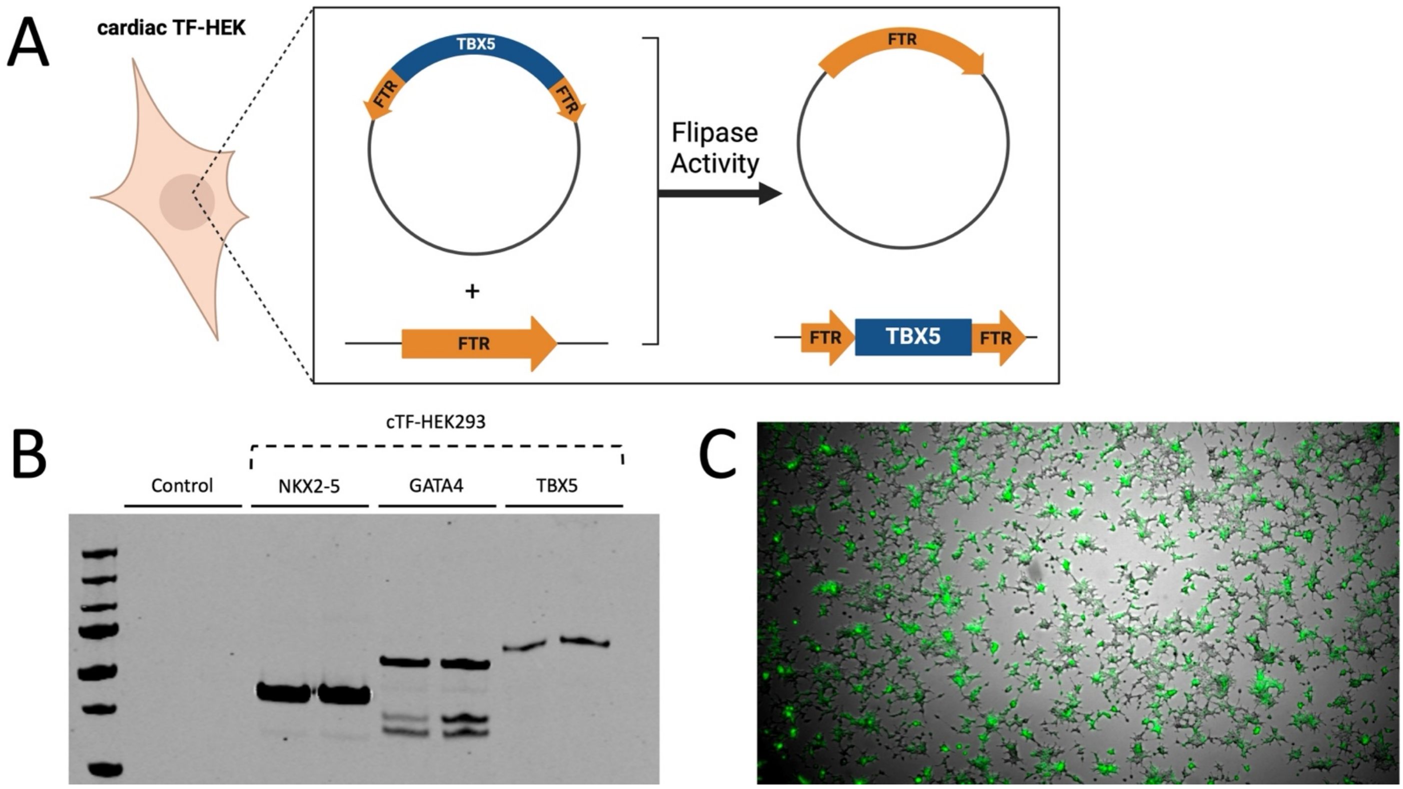

Supplementary Figure 10: Generating a cardiac TF stably-expressing HEK293 cell line. **A)** Diagram of the HEK293 FlpIn system to integrate the cardiac TF gene into the genomic landing pad. **B)** Confirmation of NXK2-5, GATA4, and TBX5 expression in HEK FlpIn cell line through Western Blot. **C)** GFP library mock transfection of HEK FlpIn.

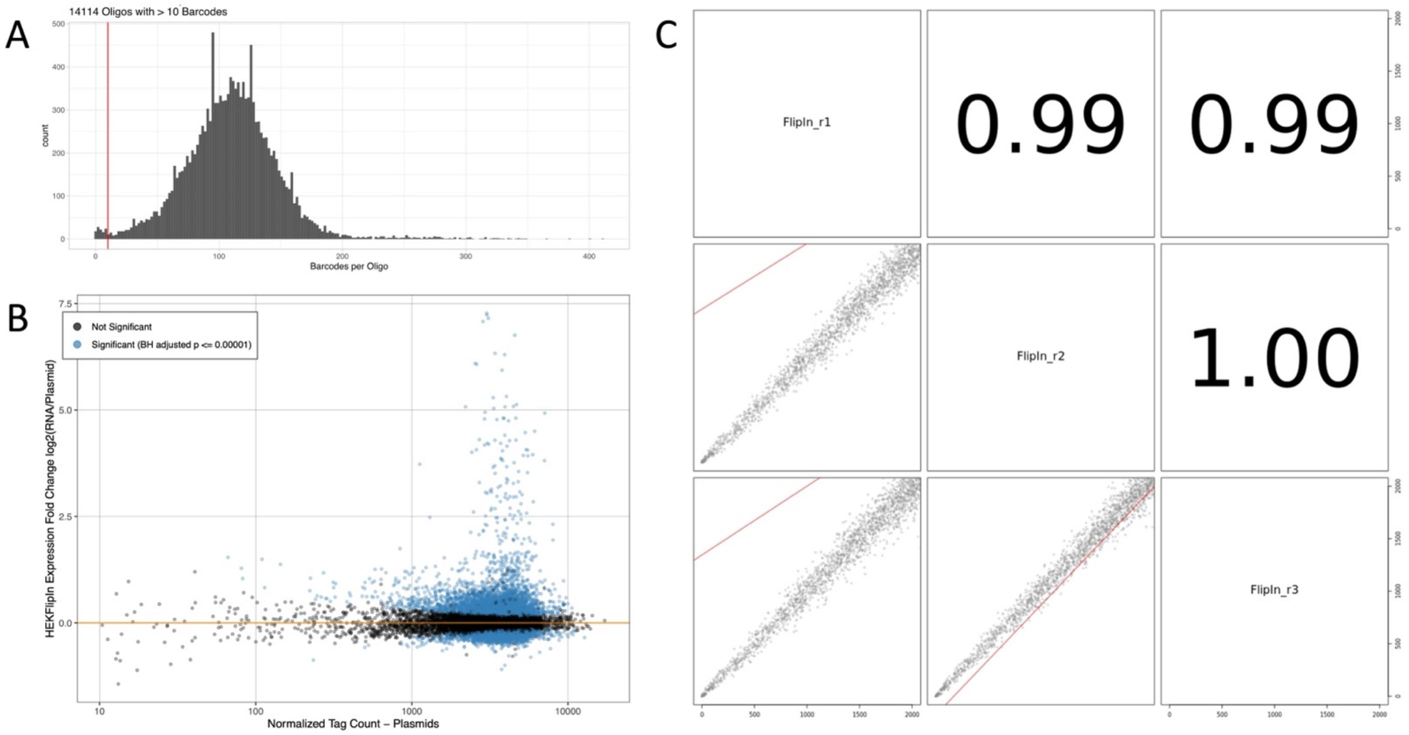

Supplementary Figure 11: MPRA quality and reproducibility analysis. **A)** Number of variants with >10 unique barcodes per oligo. **B)** Fold change of oligos compared to barcode tag counts. **C)** Correlation between biological triplicates.

	Overlap with cardiac enhancer	No overlap with cardiac enhancer	Total
**enAllele**	92	817	909
**Non enAlleles (remaining MPRA library oligos)**	295	13,057	13, 352
**Total**	387	13,874	14,261 (MPRA library)
Odds Ratio = 4.98		P-value = 2.10×10^{-29}	

	Overlap with heart DGF	No overlap with heart DGF	Total
**enAllele**	90	819	909
**Non enAlleles (remaining MPRA library oligos)**	382	12,970	13, 352
**Total**	472	13,789	14,261 (MPRA library)
Odds Ratio = 3.73		P-value = 1.57×10^{-21}	

	Overlap with both cardiac CREs	No overlap with both cardiac CREs	Total
**enAllele**	38	871	909
**Non enAlleles (remaining MPRA library oligos)**	0	13,352	13, 352
**Total**	38	14,223	14,261 (MPRA library)
Odds Ratio = infinite		P-value = 1.61×10^{-23}	

Supplementary Figure 12: Contingency tables to determine the significance of overlap between enhancer alleles (enAlleles) with cardiac regulatory elements.

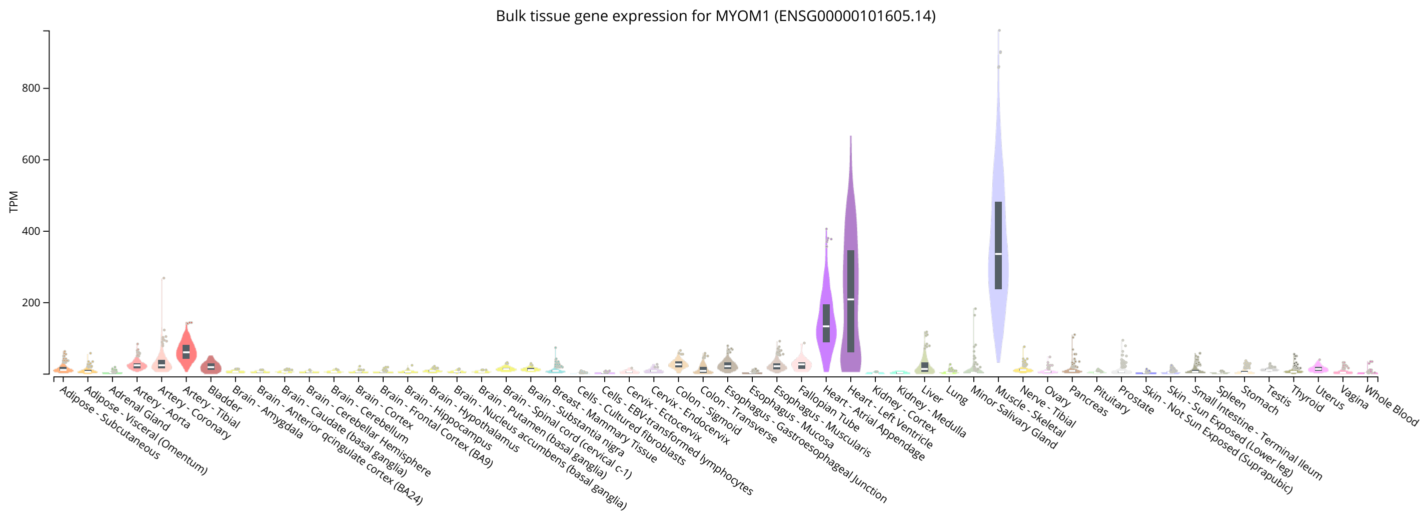

Supplementary Figure 13: MYOM1 expression profiles in multiple tissues. Cardiac tissues (the heart atrial appendage and left ventricle) are displayed in purple.

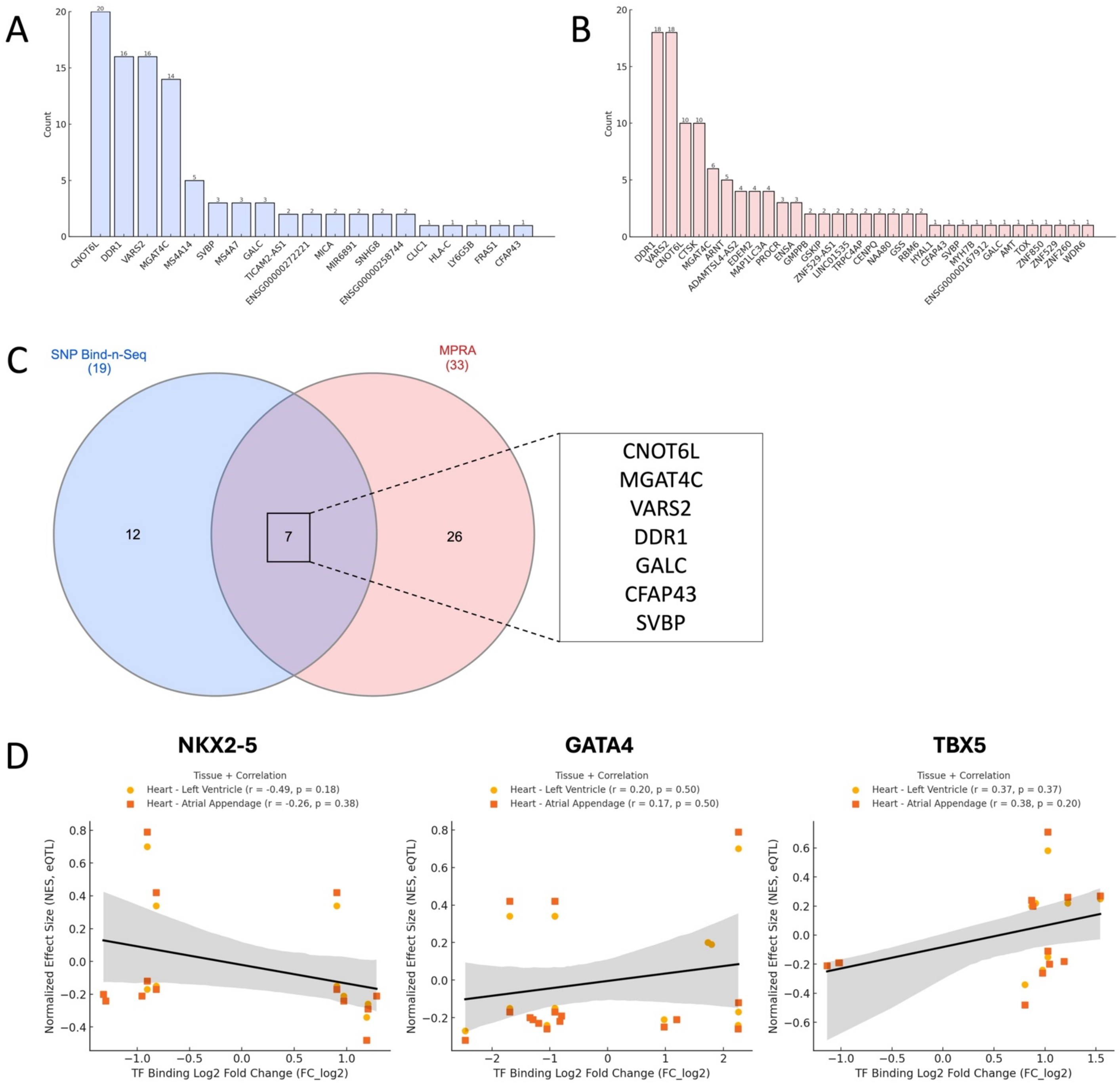

Supplementary Figure 15: Cardiac eQTL analysis of variants with genotype-dependent regulatory activity in SNP Bind-n-Seq and MPRA. **A-B)** List of genes (x-axis) and count of variants (y-axis) with cardiac eQTLS from the **A)** SNP Bind-n-Seq and **B)** MPRA experiments. **C)** Venn diagram of genes in cardiac eQTL genes from both experiments. **D**) Correlation analysis of TF binding fold change and eQTL normalized effect size.

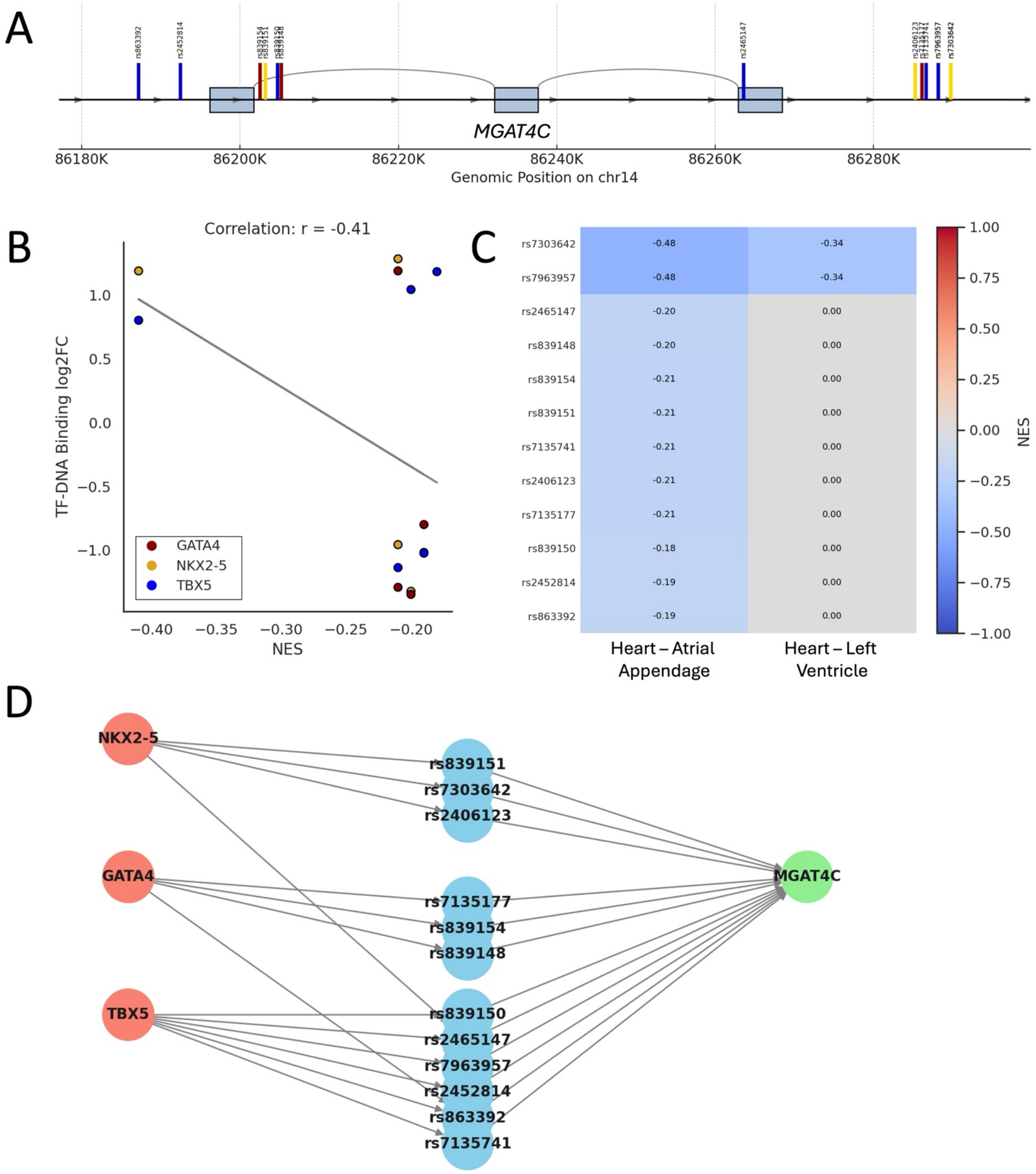

Supplementary Figure 15: Cardiac eQTL analysis of MGAT4C. **A)** Genomic map of MGAT4C with variants in cardiac eQTL. Variants are displayed as colored lines if they altered NKX2-5 (yellow), GATA4 (red), and TBX5 (blue) binding. **B)** Correlation analysis of cardiac eQTL NES and TF binding fold change. **C)** Heatmap of NES of each variant per tissue. **D**) Interaction network of cardiac eQTL genes, variants, TF with altered binding.

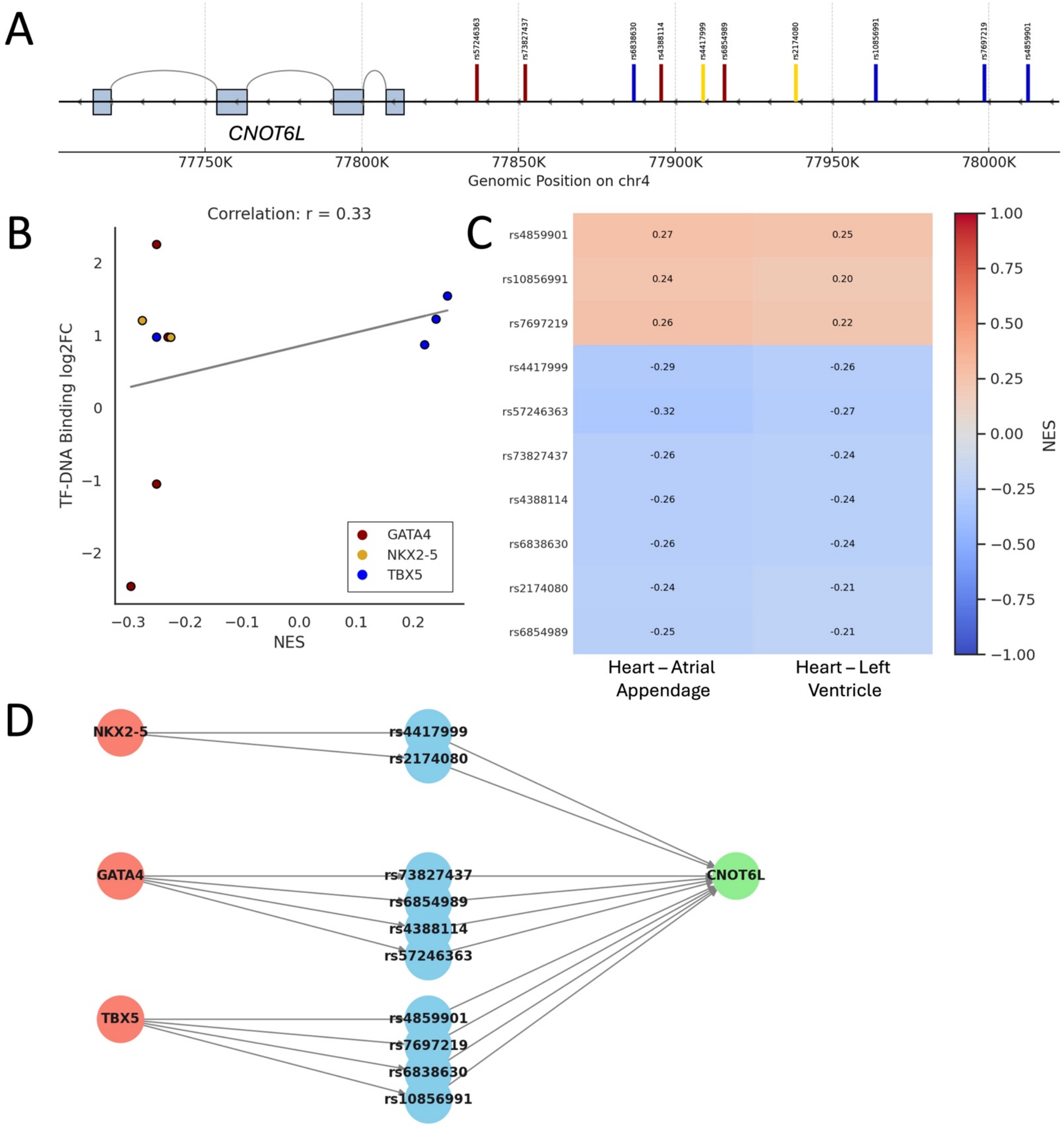

Supplementary Figure 16: Cardiac eQTL analysis of CNOT6L. **A)** Genomic map of MGAT4C with variants in cardiac eQTL. Variants are displayed as colored lines if they altered NKX2-5 (yellow), GATA4 (red), and TBX5 (blue) binding. **B)** Correlation analysis of cardiac eQTL NES and TF binding fold change. **C)** Heatmap of NES of each variant per tissue. **D**) Interaction network of cardiac eQTL genes, variants, TF with altered binding.

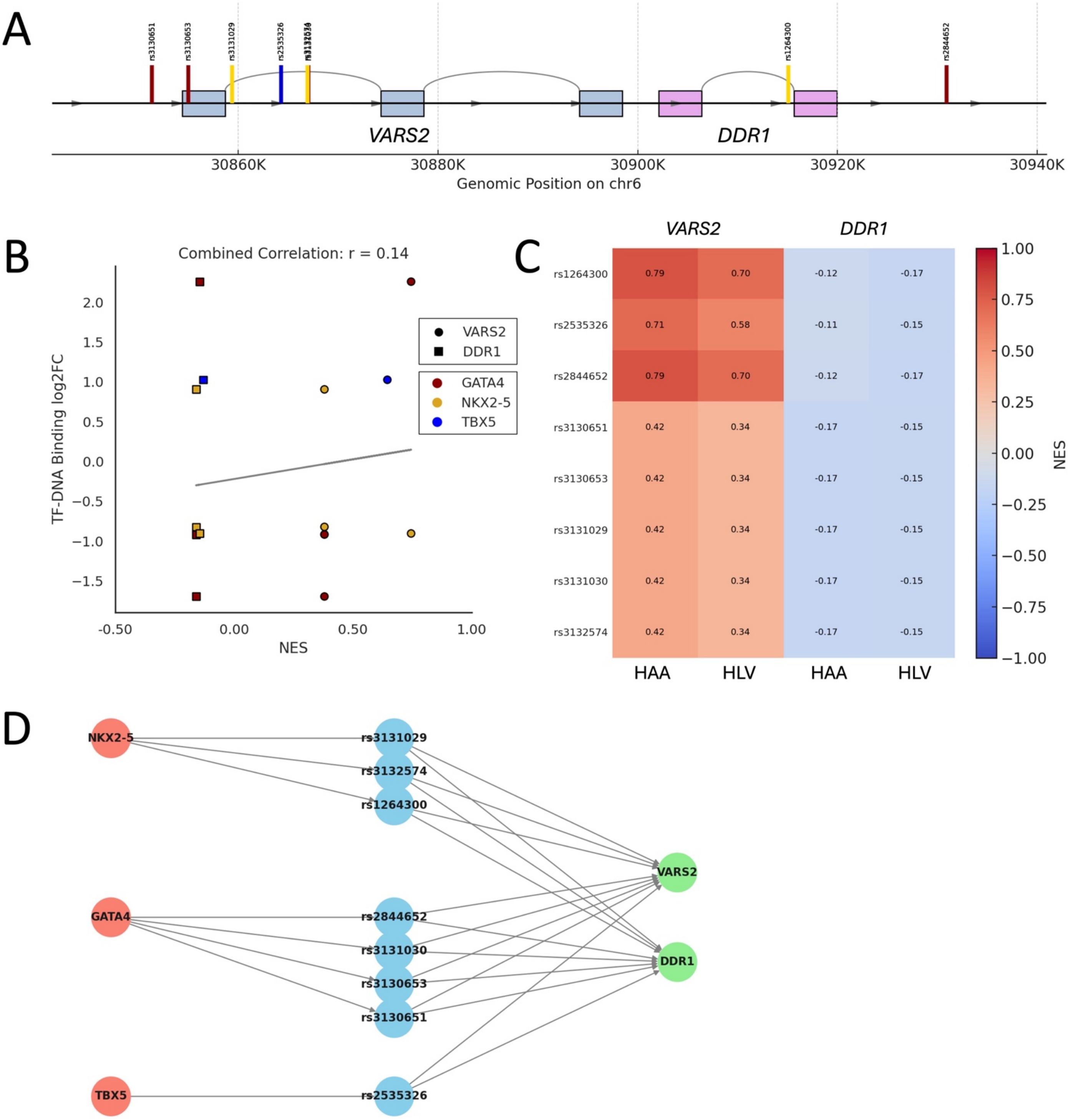

Supplementary Figure 17: Cardiac eQTL analysis of VARS2 and DDR1. **A)** Genomic map of MGAT4C with variants in cardiac eQTL. Variants are displayed as colored lines if they altered NKX2-5 (yellow), GATA4 (red), and TBX5 (blue) binding. **B)** Correlation analysis of cardiac eQTL NES and TF binding fold change. **C)** Heatmap of NES of each variant per tissue. **D**) Interaction network of cardiac eQTL genes, variants, TF with altered binding.
